# Tobacco and end stage renal disease: a multicenter, cross-sectional study in Argentinian Northern Patagonia

**DOI:** 10.1186/s12971-015-0051-x

**Published:** 2015-09-01

**Authors:** Maria M. Alba, Alicia N. Citarelli, Fernanda Menni, Maria Agricola, Alejandra Braicovich, Eduardo De Horta, Fernando De Rosa, Graciela Filanino, Raul Gaggiotti, Nelson Junqueras, Sandra Martinelli, Adriana Milan, Mabel E. Morales, Silvia Setti, Daniel O. Villalba

**Affiliations:** Northern Patagonia Association of Nephrology, Entre Ríos 651, Neuquén, 8300 Argentina; Department of Economy and Statistics of INTA (National Institute of Agrological Technology) Estación Experimental Alto Valle del Río Negro, Ruta Nacional 22 Km 1190, 8332 Allen Río Negro, Argentina; Unidad Renal Cipolletti, España 885, Cipolletti 8332 Río Negro, Argentina

**Keywords:** Chronic kidney disease, Dialysis, Tobacco renal damage, Cardiovascular diseases

## Abstract

**Background:**

Smoking and chronic kidney disease are major public health problems with common features -high prevalence and mortality, high cardiovascular risk, gender differences and high prevalence in low income people-, but the link between them is poorly recognized. Our objectives were to investigate the exposure of dialysis patients to tobacco and to know their smoking behavior.

**Methods:**

We performed a multicenter, cross-sectional study in nine dialysis units in the Argentinian Northern Patagonia. We investigated smoker status, lifetime tobacco consumption, current tobacco use, breath carbon monoxide and % carboxyhaemoglobin. Fagerström and Richmond tests were performed for active smokers. Statistical analysis: one way ANOVA and Tukey’s test for post hoc test. For exploratory analysis, frequency tables through chi-square distribution and single correspondence analysis were performed.

**Results:**

Six hundred thirty six patients (60.9 % males, 39.1 % females) were interviewed. Almost 70 % of them had had tobacco exposure. Excluding light smokers, the lifetime consumption was significantly different (*p* = 0.0052) between sexes (33.1 ± 2.4 pack/years in males and 18.2 ± 2.1 pack/years in females) The distribution of etiologies changed significantly (*χ*^2^*p* < 0.0001) with smoker status and the dose of tobacco smoking, with an increase in the diagnosis of nephrosclerosis in patients with high and very high lifetime consumption (from 16.1 % in non-smokers to 28.2 and 27 % respectively), and in passive smokers (from 16.1 to 27.3 %). The male preponderance of end-stage renal disease disappeared when only non-smokers were considered and grew with the increase in the lifetime consumption. Active smokers have small consumption, both low CO level and % COHb, low dependence and a good motivation to quit, but a high lifetime consumption.

**Conclusions:**

Exposure of dialysis patients to tobacco is high and could be related to the progression to the final stage of the renal disease. It seems that tobacco renal damage is mostly hidden in the diagnosis of nephrosclerosis. The gender difference observed in these patients could also have a nexus with the men’s higher tobacco exposure. Active smokers have a low current consumption but a high lifetime tobacco dose.

## Resumen

**Introducción:**

Tabaquismo y enfermedad renal crónica son importantes problemas de salud pública que comparten: alta prevalencia, alta morbi-mortalidad, alto riesgo cardiovascular, diferencias de género y mayor prevalencia en personas de bajos ingresos. Sin embargo, el nexo entre ellas es poco reconocido. Objetivos: mensurar la carga tabáquica de los enfermos en diálisis y conocer su patrón de consumo.

**Material y métodos:**

Participaron nueve unidades de diálisis de la Norpatagonia Argentina. Investigamos condición de fumador, carga tabáquica y, en fumadores activos, consumo actual, tests de Richmond y Fagerström, monóxido de carbono en aire espirado y % de carboxihemoglobina. Análisis estadístico: ANOVA de una vía y test de Tukey para análisis post hoc. En el análisis exploratorio, utilizamos tablas de frecuencias a través de la distribución Ji cuadrado y análisis de correspondencia simple.

**Resultados:**

Seiscientos treinta y seis pacientes (60.9 % varones, 39.1 % mujeres) fueron encuestados. Casi un 70 % de ellos había estado expuesto al tabaco. Excluyendo los fumadores leves, la carga tabáquica (CT) fue 33 ± 2.4 paquetes/año en hombres y 18.2 ± 2.1 paquetes/año en mujeres (*p* = 0.0052). La distribución de las etiologías de ingreso a diálisis cambió significativamente (*χ*^2^*p* < 0.0001) según el estado de fumador y la CT, con aumento en el diagnóstico de nefroesclerosis en fumadores pasivos (de 16.1 % en no fumadores a 27.3 %) y en pacientes con elevadas CT (de 15.2 y 16 % en CT leve y media a 28.2 y 27 % en CT alta y muy alta). La preponderancia masculina de la población desapareció en no fumadores y creció con el incremento en la CT (*χ*^2^*p* < 0.0001). Los fumadores activos tienen bajo consumo, bajo nivel de CO y carboxihemoglobina, baja dependencia y están bien motivados para dejar, pero tienen una elevada CT.

**Conclusiones:**

La alta CT de los enfermos en diálisis podría generar o contribuir a la progresión de la enfermedad renal crónica. El daño renal por tabaco se esconde principalmente en el diagnóstico de nefroesclerosis y se relaciona con la CT. La diferencia de género de estos pacientes podría relacionarse con su exposición al tabaco. Los fumadores activos tienen bajo consumo pero elevada CT.

## Background

Chronic kidney disease (CKD) is now a worldwide public health priority [1], not only for the increasing tendency but also for the high risk for cardiovascular(CV) complications related to renal function loss. CV disease is 10–20 times higher in end-stage renal disease (ESRD) patients and their most important cause of death [2]

Smoking is another major public health problem associated with CV and renal disease in the long term [3, 4]. However, around 15 % of incident dialysis patients smoke and over 60 % report previous tobacco use [5]. Smoking and CKD have common features: high prevalence [6, 7], high mortality [8, 9] high cardiovascular risk [10, 11] gender differences [12, 13] and both of them are linked to poverty [14, 15].

However, the nexus between both diseases has been underestimated, neglected or poorly recognized in nephrologycal and tobacco fields. On the other hand, the continued growth of the ESRD population around the world has been related to the underrecognition of earlier stages of CKD and risk factors for their development [16] such as hypertension, diabetes, obesity and smoking [17].

Clearly, the demographics of dialysis population has changed dramatically since the start of chronic dialysis in three essential aspects: etiology, incident age and presence of comorbid conditions. In the seventies, chronic glomerulonephritis and pielonephritis were the two more frequent causes of entry to renal replacement therapy as shown in registries [18] of that time. In fact, both represented 75 % of the total dialysis population and, surprisingly, the “microscopic renal vascular disease” (nephrosclerosis) represented only 3.5 % of etiologies and “diabetic glomerulonephritis” appeared in the list of “rarer diseases”. Back then, the majority of patients were 20–54 years old when treatment commenced. On the contrary, in the last years, the leading causes of ESRD are diabetes and nephrosclerosis, [19] the mean age increased about a decade [20] and comorbid conditions rose dramatically [21]. In Argentina, it is more and more frequent to see incident dialysis patients with several previous vascular interventions (by-pass surgery, stenting, angioplasty) or comorbid conditions directly related to the smoking status (kidney, urinary tract or lung carcinomas). Also, it is usual to see patients smoking outside of the dialysis units while they are waiting for the dialysis session to begin or the arrival of the transfer vehicle to return home.

When in 2011 we analyzed our first results about the prevalence of smoking in dialysis units of Northern Patagonia Association of Nephrology (Abstract XVII Argentinian Congress of Nephrology), we were surprised by the high number of patients (75 %) with some history of tobacco exposure.

The purpose of this research work was to measure the exposure to tobacco of ESRD patients in Argentinian Northern Patagonia in March-April 2013 and to get to know their pattern of tobacco use.

## Methods

A multicenter, cross-sectional study was conducted in March-April 2013 to assess the smoking history and habits of ESRD patients in Argentinian Northern Patagonia. The thirteen dialysis units in the “Comahue region” were invited to participate in the study but only nine responded. The survey consisted of a questionnaire in order to know smoking status, lifetime consumption, current tobacco use, motivation to stop, nicotine physical dependence and history of other addictions. The two principal investigators visited each dialysis unit and interviewed patients face to face during the hemodialysis session or during the monthly control in the case of peritoneal dialysis patients. Medical records were reviewed to obtain information about time on dialysis and the diagnosis of renal disease. All patients interviewed were briefly informed about the risks of smoking (focus on CV, vascular access and transplant risk) at the end of each interview and were offered counseling to stop in the case of active smokers. Also, the workers of the centers were informed about risks and trained in how to stimulate patients to stop.

### Smoking status

Participants were divided into four groups: nonsmokers (those who had never smoked or had smoked under 100 cigarettes in their life), past smokers (those who had smoked more than 100 cigarettes in their life and had stopped smoking at least one year before the study entry), current smokers (who smoked at the moment of interview and had smoked at least 100 cigarettes in their life) and passive smokers (people exposed to environmental tobacco for at least 10 years).

### Lifetime tobacco consumption (LTC)

It was estimated in pack-years. Pack-years were calculated by dividing the mean number of cigarettes smoked a day by 20 and multiplying with the number of smoking years. The LTC was calculated in current and past smokers and considered light if < 5 pack/year, moderate if 5–15 pack years, heavy if 16–25 pack-years and very heavy if > 25 pack-years.

### Current smokers

The smokers’ clinical history was performed for each active smoker including the Fargeström and Richmond tests as well as the determination of breath carbon monoxide in parts per million (ppm CO) with the piCO^+^ Smokelyzer. Blood carboxyhaemoglobin in percentages (% COHb) was calculated with the formula CO ppm x 0.16.

### Statistical analysis

Data was analyzed with InfoStat/P v.2013 [22] Results are shown as mean ± SEM for continuous variables, and number and percentages for categorical variables. Comparison between variables was carried out through one way ANOVA and the post hoc test used was Tukey’s testing. In order to determine the relation between variables, frequency tables through chi-square distribution were carried out. Besides when a relation did exist, single correspondence analysis was performed. Significant level was considered p-values <0.05.

## Results

### Baseline characteristics

Six hundred thirty six patients (60.9 % males, 39.1 % females) from nine dialysis units in Argentinian Northern Patagonia (Comahue region) were interviewed. This region includes two provinces, Río Negro and Neuquén, with 1,189,911 inhabitants, according to the last population census in 2010 (INDEC) [23] In both provinces, in December 2012, there were 1,185 patients under renal replacement therapy according to the Argentinian Register of Chronic Dialysis [19]; therefore, our sample included more than half of this dialysis population.

These patients comprised 98 % of the total available population (hospitalized patients or those who did not attend the dialysis session the day of the survey, did not take part in the study and only one patient refused to respond) Their mean age was 57.3 ± 0.6 years. The mean time on renal replacement treatment was 68.4 ± 2.5 months and the treatment modality was hemodialysis in 596 patients and peritoneal dialysis in 40 patients. The etiologies of end-stage renal disease were: glomerulonephritis (GN) 18 %, nephrosclerosis (NS) 19.2 %, obstructive uropathy (OU) 6.1 %, diabetes (DBT) 22.5 %, unknown (UK) 22.6 % and others (OT) 11.5 %.

### Smoking status (SS)

There were 93 (14.6 %) current smokers and 543 (85.4 %) non- current smokers. In the last group, 269 (49.5 %) were never smokers, and 274 (50.5 %) were former smokers. But in the group of never smokers there were 77 (28.6 %) passive smokers (past or present). Therefore, almost 70 % of the population had had tobacco exposure.

### Lifetime tobacco consumption (LTC)

It was calculated in current and past smokers and was very high (>25 pack/years) in 24.4 %, high (16–25 pack/years) in 10.2 %, moderate (5–15 pack/years) in 21.6 % and light (<5 pack/years) in 43.7 %. Excluding light smokers, the mean LTC was 33.1 ± 2.4 pack/years in males and 18.2 ± 2.1 pack/years in females (*p* = 0.0052). The distribution of etiologies varied significantly (*χ*^2^*p* < 0.0001) according to smoker status (Table 1) and LTC (Table 2)

**Table 1:** Differences in etiology distribution according to smoker status

End-stage renal disease etiology	Smoker status				Total (number and %)
	Current (% and 95 % CI)	Never (% and 95 % CI	Passive (% and 95 % CI	Former (% and 95 % CI	
Diabetes	14 (6–21.9)	24.5 (20.6-28.3)	22 (21.7-22.4)	24 (19.7-28.5)	143 (22.5)
Glomerulonenephritis	28 (18.8-37.1)	18.2 (17.9-18.5)	13 (9–16.9)	16 (10.5-21.6)	115 (18.1)
**Nephrosclerosis**	19.3 (19.2-19.5)	**16.1 (10.3-21.9)**	**27.3 (21–33.5)**	19 (18.4-19.5)	122 (19.2)
Unknown	23.7 (22.7-24.6)	25.5 (19.9-31)	16.9 (12.4-21.3)	22 (19.8-23.9)	144 (22.6)
Obstructive uropathy	0	5.7 (4.9-6.5)	5.2 (4.4-5.9)	8.8 (1.5-15.9)	39 (6.1)
Others	15 (11.7-18.3)	10 (6.8-12.9)	15.6 (12.4-18.7)	10.2 (6.7-13.6)	73 (11.5)
Total (number and %)	93 (14.6)	192 (30.2)	77 (12.1)	274 (43.1)	636 (100)

The change in etiology distribution achieved statistical significance (*χ* ^2^ = 82.34; *p* < 0.0001), with an important increase (from 16.1 % to 27.3 %) in nephrosclerosis in passive smokers (bold text)

**Table 2:** Etiologies and lifetime tobacco consumption in past and current smokers

Etiology of end-stage renal disease	Lifetime tobacco consumption (% and 95 % CI)				Total (number and %)
	Light <5 pack/years	Medium 5–15 pack/years	High 16–25 pack/years	Very high >25 pack/years	
Diabetes	19.6 (16.6-22.6)	24.7 (22.1-27.2)	15.4 (13–17.8)	24.7 (21.9-27.6)	79 (21.5)
Glomerulonephritis	23.4 (16.5-30.2)	19.8 (19.2-20.3)	12.8 (10.4-15.3)	13.5 (8.5-18.5)	70 (19.1)
**Nephrosclerosis**	**15.2 (8.2-22.2**	**16 (13.2-18.9)**	**28.2 (24.9-31.6)**	**27 (20.4-33.5)**	72 (19.6)
Unknown	23.4 (20.8-26)	19.8 (18.1-21.4)	28.2 (25.7-30.7)	18 (14.6-21.4)	80 (21.8)
Obstructiveuropathy	6.3 (5.6-7)	3.7 (1.2-6.2)	2.6 (0.9-4.2)	12.4 (7.4-17.3)	25 (6.8)
Others	12 (10.7-13.4)	16 (12.1-20)	12.8 (12.2-13.5)	4.5 (1.45-10.4)	41 (11.2)
Total (number and %)	158 (43)	81 (22)	39 (11)	89 (24)	367 (100)

Lifetime tobacco consumption (LTC) was calculated in pack-years. The change observed in etiology distribution achieved statistical significance (*χ* ^2^ = 42.23; *p* < 0.0001). The diagnosis of nephrosclerosis increased from 15.2 % and 16 % in patients with light and medium LTC to 28.2 % and 27 % in patients with high and very high LTC (bold text)

.

The diagnosis of nephrosclerosis increased in all categories of smoker but passive smokers showed a more important increase (from 16.1 % in non-smokers to 27.3 %). In smokers with high and very high LTC, the percentages grew from 15.2 and 16 % in light and medium LTC to 28.2 and 27 % respectively.

### Gender

Sex distribution differs significantly (*χ*^2^*p <* 0.0001) according to SS (Table 3)

**Table 3:** Gender and smoking status

Gender	Current smokers (% and 95 % CI)	Current non smokers (% and 95 % CI)		
		Never	Passives	Former
Men (number = 387)	**74.2 (61.2-86**.6)	**45.8 (45.8-74.6)**	29.9 (6–53.7)	**75.5 (35.3-115.8)**
Women (number = 249)	25.8 (13.4-38.2)	54.2 (54.2-83)	**70.1 (46.3-94)**	24.5 (15.8-64.7)
All (number = 636)	93	192	77	274

Sex distribution differed significantly (χ^2^81.84; *p* < 0.0001) according to smoking status. The male preponderance of ESRD disappeared when we considered only never smokers and grew if we focused on former and current smokers; in passive smokers there was prominent female majority (bold text)

.

In never smokers it was 54.2 % for women and 45.8 % for men, whereas in current and former smokers we observed male preponderance (75 %). If we consider only current and former smokers with high and very high LTC, the percentage rose to 85 %. Instead, female preponderance was observed in passive smokers. We also made a simple correspondence analysis to observed associations between age, gender and smoker status. Active smokers were younger than former smokers. Male gender was associated to past smoker and age over 40 years whereas female gender was related to non-smoker and passive smoker.

### Current smokers

17.8 % of males and 9.6 % of females were smokers (*n* = 93). Their average age was 48.3 ± 1.6 years, of which 74.2 % were males and 25.8 % were females. Current tobacco use was <10 cigarettes/day in 68.8 % of cases, between 11–20 cig/d in 15 %, more than 20 cig/d in 5.3 % and there were another 10.8 % of patients with recent quitting (less than one year) that we cannot yet consider former smokers. Only one patient consumed more than 31 cig/d. LTC was very high in 35 smokers, high in eight, moderate in 25 and light in the other 25. According to Richmond test, 26.9 % of patients were in the pre-contemplation stage, 39.8 % in the contemplation stage and 22.5 % in preparation phase (high motivation for quitting). According to Fagerström test, dependence was low in 90.3 %. Other addictions were informed in 24 cases (alcohol, analgesic or illegal drugs). Average level of breath CO was 10.8 ± 0.8 ppm and % COHb 1.7 ± 0.1.

## Discussion

According to our investigation, it seems that dialysis units are nowadays bastions of heavy smokers (past or current) surviving thanks to modern medical advances. They have a low current consumption but an important lifetime consumption which might have influenced the progression to ESRD and could contribute to the gender difference observed in this disease. Also the exposure to second hand smoke could have a nexus with chronic kidney disease.

The suspicion that smoking could be a kidney risk factor dates back to the first half of the 20th century [24] but it took many years for the evidence supporting this suspicion to make itself present. Smoking was pointed out for the first time as a renal risk factor 35 years ago (1978) when Christiansen [25] reported a more rapid progress of diabetes nephropathy in smokers, and Dales [26] observed that the presence of proteinuria was more common in smokers and more prominent in heavy smokers. In the final years of the 20th century, prestigious nephrologists [27] highlighted something that cardiologists, neurologists and vascular surgeons had concluded many years earlier: that smoking was a vascular risk factor and, as a consequence, an important renal risk factor such as diabetes or hypertension which, basically, induce renal damage by means of vascular damage. However, for reasons difficult to understand, nephrologists couldn’t accept something that seemed obvious if we consider that the vascular renal network is one of the widest of the body [28]. Perhaps, the reason for this omission is related to the silent characteristics of renal disease: there isn’t “renal angina”, the “vascular renal accident” is diagnosed only exceptionally, and the loss of renal function does not generate dramatic situations such as a myocardial infarction, a cerebral vascular accident, a member amputation or sexual disfunction. On the other hand, the delayed nephrology consultation leads to a significant number of patients starting renal replacement therapy without accurate diagnosis.

The tobacco exposure of our patients was similar to that reported by Banas et al. [29] in patients on the waiting list for renal transplant (70 % of patients with tobacco exposure). This observation confirms our suspicion that there is more tobacco exposure in dialysis patients than in the general population, where the exposure reaches 50 % [30] In that survey, the percentage of current smokers was higher (24 %) than in our investigation (14.6 %). Perhaps, this difference is a result of the fact that patients on the waiting list are younger, with less comorbidities, and are healthy enough to be able to smoke. Our prevalence of passive smokers was higher (28.6 %) than what was reported on the general population (12 %) [31] but very similar to what was reported in patients with coronary heart disease [32]. Unfortunately, in the Banas study, there was not information about these smokers.

The mean LTC found in our patients was higher than that reported by Banas (33.1 pack-years for men and 18.2 pack-years for women vs 17 and 14.5 pack-years respectively), but our analysis excluded smokers with <5 pack-years and this could be the reason for the difference. Moreover, our study included all dialysis patients and the Banas study included only waiting list patients.

The etiologies of ESRD were comparable with those reported by the ARCD. Three etiologies, diabetes (23 %), nephrosclerosis (19 %) and unknown (23 %) lead the register including 65 % of prevalent dialysis patients. However, when we focus on passive smokers and smokers with high and very high LTC, there is an important increase in the diagnosis of NS, suggesting that in this diagnosis the heaviest tobacco burden is hidden. In a survey on Swedish population, Ejerblad et al. [33] informed about the increase in the diagnosis of NS with the increase in LTC. We want to highlight the change observed in passive smokers because this feature is very important if we consider the reported relationship between passive smokers and vascular damage [34, 35]. Vascular, interstitial and glomerular lesions has been described in long-term smokers but vascular damage is preponderant [36, 37]. Cigarette smoke damages endothelial cells and nicotine induces smooth muscle cell proliferation [38]. Our study is the first to inform about the exposure to second hand smoke in ESRD patients.

The incidence and prevalence of ESRD is greater in men than in women [39] but the underlying mechanisms are not clearly identified. The factors involved may include diet, glomerular and kidney size, differences in glomerular hemodynamics or sex hormonal effects [40]. When we analyzed only never-smoking patients, the male preponderance disappeared, but it grew when we focused on smokers (past and current), and grew even more with the increase in tobacco exposure. When we looked at the 2012 ARCD [19], we were surprised by the fact that there are no gender differences until the age of 45, but in the subsequent decades, the gender difference becomes increasingly prominent. This leads us to think that the difference may be caused by a factor that takes a long time to cause damage (tobacco?). Up to now there is not any survey that has investigated the role of tobacco in the greater prevalence of CKD on men, thus opening an interesting research field.

Finally, current smokers were about a decade younger than the total population with a relevant male preponderance (74.2 % men). In most of them, the actual consumption was low but LTC (>5 pack-years in 70 %) was high. However, it has been reported that many persons quit smoking only after the onset of severe illness [41] Indeed, 10.8 % of our patients had quit recently as a consequence of the impact of starting dialysis, as was pointed out by Banas et al. These patients gave us an excellent opportunity to encourage quitting, focusing on the specific benefits to quit [42] -lower obstruction rate of vascular access [43], lower cardiovascular risk [44] and better post- transplant evolution [45].

The Richmond test [46] was designed to know the readiness of the smoker to stop smoking. Prochaska and Di Clemente [47] developed the “stage of change model” for assessing it. About 40 % of smokers in the general population are not ready to quit and another 40 % are “unsure”. Only 20 % of smokers are ready to stop. In the case of our patients, if we add 22.5 % of patients ready to stop to 10.8 % of patients with recent quitting, we have 33.3 % of patients with a good motivation to stop, which outnumbers the reported percentages of these change steps in the general population [48].

The Fagerström test [49] evaluates tobacco physical dependence. When physical dependence is high, consumption is also high and heavy smokers start to smoke early in the morning in order to increase their falling nicotine levels after night deprivation. Instead, smokers with ESRD have a small consumption that usually starts after midday and is influenced by the dialysis treatment: smokers with ESRD smoke before the dialysis treatment to relax and start smoking again very soon after the dialysis treatment. If we consider that these patients have smoked for many years and most of them have just had complications related to tobacco, it seems difficult to believe that they have “low dependence”. We think that our patients may have tobacco psychological dependence that unfortunately we have not investigated, and maybe, the Fagerström test might require some changes for these particular patients.

Breath carbon monoxide is a risk marker for the development of diseases related to tobacco and a valid indirect marker of carboxyhaemoglobin level, with a linear relationship between them [50]. The mean level of carbon monoxide of our patients (10.8 ppm) was a little over the cutoff point of 8 ppm between smokers and non-smokers [51] according to the low consumption reported by the majority of them. We observed a similar feature in the percentage of carboxyhaemoglobin (1.7 %) which has a cutoff point of 1.66 % according to Jarzon et al. [52] However, these levels could be dangerous in the context of ESRD patients, frequently anaemic, with an extremely high cardiovascular risk.

Some smokers, such as smokers with COPD [53] have specials patterns of tobacco consumption (higher consumption, higher dependence and higher CO in exhaled air). Instead, our dialysis patients have a small current consumption, a low level of CO, low dependence but an important LTC. Up to now, there has been no information about the smoking habits of ESRD patients and our study is the first to explore them.

The personal contact between patients and the two main investigators (especially trained in tobacco control) is the greatest strength of our study. On the contrary, the main limitations lie in the absence of a non-renal control population, the limited sample size and the presence in CKD patients of many confounding factors. Unfortunately, we were not able to demonstrate associations between LTC and non- renal complications linked to tobacco use because not all patients had been screened in the same way (it was a multicenter, cross-sectional study). However, if we add the present results to the cumulative evidence since the start of this century, it seems absolutely necessary to enlarge this cohort in order to achieve major statistical power.

## Conclusions

The high exposure of dialysis patients to tobacco (past, passive or current) could be related to the increasing worldwide tendency of end stage renal disease and to the gender difference observed in this disease. The diagnosis of nephrosclerosis seems to hide the most important tobacco burden. Active smokers have low dependence, good motivation to quit, low actual consumption but high lifetime tobacco consumption. Nephrologists and tobacco specialists should work together to find a marker of “tobacco nephropathy” that permits their inclusion as an etiologic cause of ESRD.
